# The Use of Chitosan-Coated Nanovesicles in Repairing Alcohol-Induced Damage of Liver Cells in Mice

**DOI:** 10.3390/medicina58060762

**Published:** 2022-06-05

**Authors:** Loredana Nicoleta Hilițanu, Liliana Mititelu-Tarțău, Maria Bogdan, Beatrice Rozalina Buca, Ana-Maria Raluca Păuna, Liliana Lăcrămioara Pavel, Ana-Maria Pelin, Andreea-Daniela Meca, Grațiela Eliza Popa

**Affiliations:** 1 Department of Pharmacology, Faculty of Medicine, “Grigore T. Popa” University of Medicine and Pharmacy, 700115 Iasi, Romania; ln.rusu@yahoo.com (L.N.H.); beatrice-rozalina.buca@umfiasi.ro (B.R.B.); paunaanamariaraluca@gmail.com (A.-M.R.P.); 2 Department of Pharmacology, Faculty of Pharmacy, University of Medicine and Pharmacy, 200349 Craiova, Romania; andreea_mdc@yahoo.com; 3 Department of Morphological and Functional Sciences, Faculty of Medicine and Pharmacy, “Dunărea de Jos” University, 800010 Galați, Romania; doctorpavel2012@yahoo.com; 4 Department of Pharmaceutical Sciences, Faculty of Medicine and Pharmacy, “Dunărea de Jos” University, 800010 Galați, Romania; anapelin@gmail.com; 5 Department of Pharmaceutical Technology, Faculty of Pharmacy, “Grigore T. Popa” University of Medicine and Pharmacy, 700115 Iasi, Romania; eliza.popa@umfiasi.ro

**Keywords:** alcohol, chitosan, hepatoprotective, lipid vesicles, mice

## Abstract

*Background and Objectives* In the past few decades, the studies concerning the natural polysaccharide chitosan have been centered on a new direction: its hepatoprotective action. The aim of our study was to evaluate the influence of previously designed chitosan lipid vesicles on the liver damage induced by alcohol consumption in mice. *Materials and Methods* The study involved the oral administration of substances in one daily dose as follows: Group 1 (control): water; Group 2 (control alcohol): 5% alcohol in water; Group 3 (CHIT): 0.1 mL/10 g body weight chitosan solution in animals treated with alcohol; Group 4 (CHIT-ves): 0.1 mL/10 g body chitosan vesicles in animals treated with alcohol; Group 5 (AcA): 200 mg/kg body ascorbic acid in animals treated with alcohol. In order to evaluate liver damage after alcohol consumption, the following hematological parameters were tested: the activity of alanine aminotransferase, aspartate aminotransferase and lactate dehydrogenase; serum values of urea and creatinine; the phagocytic capacity of polymorphonuclear neutrophilsin peripheral blood;serum opsonic capacity;bactericidal capacity of peritoneal macrophages; and the activity of malondialdehyde, glutathione peroxidase, superoxide dismutase and lactate dehydrogenase. *Results* and *Conclusions* The treatment with chitosan vesicles decreased liver enzyme activity and reduced the oxidative stress disturbances in alcoholic mice, thus repairing the hepatic functional and structural damages. These beneficial activities of chitosan vesicles were comparable with ascorbic acid effects in alcoholic mice.

## 1. Introduction

Recent research has focused on the potential of various natural biocompatible compounds and biodegradable polymers as drug delivery systems [1,2]. 

**Figure 1:**
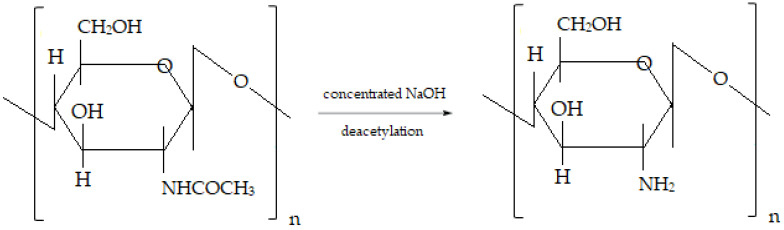
Chitin deacetylation into chitosan.

One of the most interesting drug-carrier mono-polymers is represented by a polysaccharide called chitosan, obtained from the deacetylation of chitin (Figure 1) [3], an essential structural element of arthropod exoskeletons, fungal cell walls and squid pens. The linear cationic structure of chitosan contains both units of β-(1→4)-2-amino-D-glucose and β-(1→4)-2-acetamido-D-glucose [4,5], and its positive charge furtherensuresproper binding to the predominantly negatively charged biological membranes [6].

Primary amines within the chitosan structuregive the polymer its character of cationic polyelectrolyte [7], and it isfurther able to interactwith cell membranes, as well aswith the vesicular lipid bilayer; these also properties give chitosan the ability to encapsulate active substances [8,9]. Chitosan is soluble in acidic water-based solutions, in relation to the value of the pH of the environment and the percentage of deacetylation, due to protonation of the amino groups in an environment with lower values of pH [10,11]. Chitosan may also display various properties depending on the differences in molecular mass, as well as the structural position of acetylated residues within the main chain of the polymer and the degree of deacetylation [12]. 

Moreover, chitosan has gained attention as biomedical material [13,14] due to its multifaceted activities, such as anti-inflammatory [15], antibacterial [16], antitumoral [17,18,19], immunostimulatory [20], anti-hyperglycemic and anti-hyperlipidemic [21,22], antiulcer [23] and antioxidant [24,25] activities. The literature data highlight that this polymer can be formulated into various dosage forms: solutions, gels, solid mixtures, sponges, prolonged-release tablets, pastes and nanoparticles, for different applications [26].

Nevertheless, chitosan possesses several ideal properties for obtaining carrier polymeric nanoparticles, such as efficiency, biocompatibility, biodegradability, non-toxicity and relatively low cost [27,28,29]. Moreover, chitosan nanoparticles have proven hepatoprotective activity during alcohol consumption and against the effects of lipid-rich diet [30]. Researchers underline the decrease in macrophage polarization and pro-inflammatory cytokines in the liver after administering chitosan oligosaccharides in mice [31]. By ensuring proper serum levels of reduced glutathione and glutathione reductase and peroxidase and by upregulating signalization pathways through various antioxidants in experimental studies on rats, chitosan oligosaccharideshaveproven antioxidative effects [32]. However, little information is available, to our knowledge, regarding the use of chitosan vesicles in mice with alcohol-induced hepatotoxicity as well as the relationship between lipid vesicles incorporating chitosan and liver toxicity. Agents capable of decreasing associated liver damage induced by alcohol are a promising line of research, as worldwide chronic alcohol consumption leads to high annual rates of mortality [33]. Analyzing the potential antioxidant and hepatoprotective activity of chitosan may contribute to WHO policies [33].

Alcohol exerts toxic effects on the hepatocellular level, represented by the increased serum level of transaminases following their release into the circulation, after cell destruction. At the same time, there is an intensification of oxidative stress and lipid peroxidation as a result of the alteration of proteins and lipids by free radicals resulting from hepatocyte damage.Treatment with chitosan and chitosan vesicles resulted in a significant decrease in liver enzyme activity, which may be attributed to the antioxidant and anti-inflammatory effects of this polysaccharide [30,34,35]. The repair actions of chitosan and chitosan vesicles can be explained by a possible stabilizing effect of the hepatocyte membrane, preventing the release of transaminases into the blood. The improvement of the endogenous capacity of antioxidant defense, by intensifying the activity of antioxidant enzymes and depleting the pro-oxidant ones, is an additional effect.

Therefore, the aim of our research was to perform the structural analysis of lipid vesicles with chitosan and to evaluate their influence on structural and functional changes in mice with experimentally induced alcoholic liver toxicity.

## 2. Materials and Methods

### 2.1. Substances 

Egg yolk specific type XVIE L-alpha phosphatidylcholine (99% TLC purity), chitosan (from crab shells, practical grade) and chloroform were supplied by Sigma-Aldrich Chemical Co. (Steinheim, Germany). The chitosan used had the following characteristics: degree of N-deacetylation = 28%; polydispersity index = 3.26; average molecular weight Mn = 94,810; average gravimetric mass Mw = 309.900. Chitosan was dissolved in 1% (*w/w*) acetic acid solution (in deionized water, obtained with an UltraMatic PLUS DI apparatus—Wasserlab, Navarra, Spain).

### 2.2. Chitosan Lipid Vesicles Preparation

A solution of L-alpha phosphatidylcholine was obtained by dissolving 25 mg in 1 mL chloroform, and then the solvent was evaporated in order to obtain a dry lipid film. By hydrating the dry lipid film in 50 mL deionized water with 1% (*w*/*v*) chitosan solution in 1% (*v*/*v)* acetic acid, the lipid thermodynamic instable monolayers were achieved. The monolayers were disrupted by ultrasonication for 10 min, at room temperature (10% amplitude mode, at 20 kHz ± 500 Hz standard frequency; SonoplusBandeline set-up), thus entrapping water in the lipid vesicles coated with chitosan. 

The size distribution of chitosan vesicles and the solution stability, established according to the average value of zeta potential, were assessed using a Malvern Zetasizer Nano ZS ZEN-3500 Apparatus (Worchestershire, UK).

### 2.3. Laboratory Animals 

The experiments were performed on healthy non-genetically modified white Swiss mice (aged 6–8 weeks, 25–30 g weight each), with uniform gender distribution. The animals were purchased from the ‘Cantacuzino’ National Medical-Military Institute for Research and Development, Baneasa Station, Bucharest, Romania, and brought to the bio-base of the University of Medicine and Pharmacy ‘Grigore T. Popa’ Iasi within the CEMEX Laboratory (Advanced Research and Development Center in Experimental Medicine). The accommodation of the animals took place the day before the experiment: the mice were kept in standard laboratory conditions (with constant temperature of 21 ± 2°C, relative humidity of 50–70% and alternating lighting regimen (12/12 h light/dark ratio). 

The animals received drinking water ad libitum from standardized containers and were fed with pellets. The weight of the mice was measured daily before the start of the experiments, and the amount of consumed food was determined for each one. During the experiments, the mice were not allowed to drink water or eat. To prevent chronobiological influences on animals, the experiments took place during the same hours every day (8–12 a.m.).

### 2.4. Investigation of Chitosan Vesicles Effects in Mice with Experimental Induced Alcoholic Liver Toxicity

For the experiment, the animals were randomly assigned to 4 groups of 6 mice each, to which hepatopathy was induced using ethanol; the mice received the test substances orally in once-daily administration, during the last 7 days of the experiment, as shown in Table 1:

- Group 1 (Control): water ad libitum, without alcohol;
- Group 2 (Control alcohol): 5% alcohol in water ad libitum;
- Group 3 (CHIT): 0.1 mL/10 g body chitosan solution in animals with alcoholic liver toxicity;
- Group 4 (CHIT-ves): 0.1 mL/10 g body solution containing chitosan vesicles in animals with alcoholic liver toxicity;
- Group 4 (AcA): 200 mg/kg body ascorbic acid in animals with alcoholic liver toxicity.

This experimental model of alcoholic liver toxicity induction consists in the ad libitum use of an aqueous solution containing 5% alcohol as drinking water (in special devices for animals) for a period of 2 weeks and the direct intragastric administration (using an eso-gastric tube) of alcohol 10% solution (5 g/kg body weight) in the last 3 days of the experiment (Table 1) [36,37,38]. This is an animal model that combines constant alcohol intake with acute administration, which results in elevated blood alcohol levels [39,40].

Ascorbic acid (200 mg/kg body weight) is used as a positive control in the experiment. Its protective effects on the liver are wellknown in mice with toxic liver disease induced by drugs or by various chemicals [41,42,43,44,45,46,47,48].

In order to determine how alcohol consumption causes liver and kidney damage, hepatic and renal function assessment parameters are evaluated at regular intervals to investigate in detail how alcohol consumption gradually affects liver function and structure over time [49,50].

Blood samples were collected for laboratory tests before the beginning of the experiment and on the last day of the investigation (two hours after the administration of the substances). At the end of the experiment, the animals were sacrificed to extract liver and kidney fragments for histopathological examination.

The evaluation of the influence of chitosan vesicles on liver function consisted in investigating the following parameters: the hematological profile and the activity of alanine aminotransferase (ALT), aspartate aminotransferase (AST) and lactate dehydrogenase (LDH). The integrity of kidney function was assessed by measuring blood urea and creatinine levels. We also evaluated levels of some immune parameters: phagocytic capacity of polymorphonuclear neutrophils (PMN) in peripheral blood (PC);serum opsonic capacity (OC);bactericidal capacity of peritoneal macrophages (BC); and activity of malondialdehyde (MDA), glutathione peroxidase (GPx), superoxide dismutase (SOD) and LDH. In order to perform biochemical determinations, 0.5 mL of blood was collected on an empty stomach from one of the caudal veins of the animal tail. From collection until determination, blood samples were kept in a mixture of ice and water. The hematological and biochemical parameters were assessed using the Hematology Analyzer 5 DIFF model BF-5180 (DIRUI Manufacturer).

The phagocytic capacity ofPMN in peripheral blood (PC) was determined on the 7th day of the experiment. At the end of the experiment, the animals were anesthetized with 3% isoflurane and sacrificed. Ten milliliters ofHank’ssolution (thermostated at 37 °C) wasused to wash the intact peritoneal cavity of animals in order to remove the peritoneal macrophages. The centrifugation of the samples was performed for 10 min (1000 rpm), followed by mixing with *Staphylococcus aureus* 94 strains in 0.2% glucose broth cultures. The mixtures were diluted (1:1000) in brine, allowedto incubate for 48 h at 37 °C and reseeded on culture media, displaying the formation of plaque colonies. The measured parameters of peritoneal macrophages were OC and BC.

Quantification of plasmatic MDA levels was performed spectrophotometrically based on the thiobarbituric acid method using the RANSOD kit from RANDOX Laboratories Ltd. (Warsaw, Poland) [51]. The activity of GPx in plasma was assessed spectrophotometrically with a specific RANSOD kit from RANDOX Laboratories Ltd., using the reduction procedure of nicotinamide adenine dinucleotide phosphate [52]. The RANSOD kit from RANDOX Laboratories Ltd. was used for the erythrocyte determination of SOD by the colorimetric method with xanthine and xanthine oxidase.

After sacrificing the animals, liver and kidney fragments were collected and prepared for histopathological examination. The collected tissues were fixed in 10% formalin solution. The fragments were embedded in paraffin and sectioned 5 μm apart; the staining was performed with hematoxylin–eosin (H&E). The tissue samples were observed with an optical microscope equipped with a digital camera (Nikon TI Elipse device, Nikon Coolpix 950 camera, optical zoom × 3, 1600 × 1200 resolution—1.92Mpx).

The protocol of our experimental research received the approval from the Ethics Committee from the University of Medicine and Pharmacy ‘Grigore T. Popa’ Iasi (Certificate No. 24/14 July 2020), and the tests were performed according to the specific ethical directions and to the international ethical standards on experiments on laboratory animals. The methodology of experimental investigations was established in accordance with national and international standards which refer to the protection of animals used for scientific purposes [53,54].

### 2.5. Statistical Analysis of Data

The collected data were statistically analyzed using the one-factor ANOVA method implemented in the SPSS 17.0 software for Windows 10 and were expressed as the mean values ± standard deviation (SD) of the mean. For multiple comparisons, Tukey and Newman–Keuls tests were added to the whole statistical analysis, given that post hoc tests can separate the lots by groups of significance. By means of these tests, a scale of substance effect intensity can be obtained. The significant differences among the same group of animals can be recorded, as can the significant differences between groups receiving different substances, compared to the control group. The *p* < 0.05 values were considered to be statistically significant compared to those of the control group.

## 3. Results

### 3.1. Size, Zeta Potential and SEM Microscopy of Lipid Vesicles with CHIT

**Figure 2:**
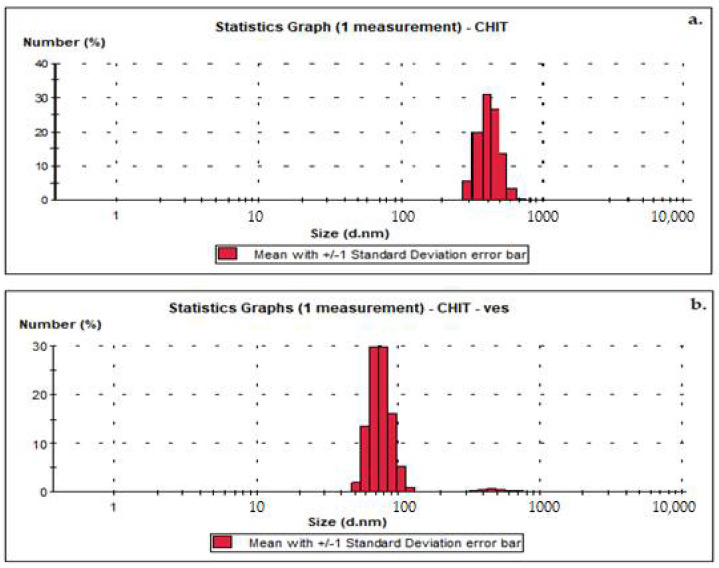
Size distribution ofchitosan solution (**a**) andchitosan vesicles (**b**).

The chitosanparticle size ranged from 458 nm to 845 nm with an average size of 613 nm (Figure 2a). The dimension determination of chitosan vesicles showed the average size distribution value of 84 nm (Figure 2b).

**Figure 3:**
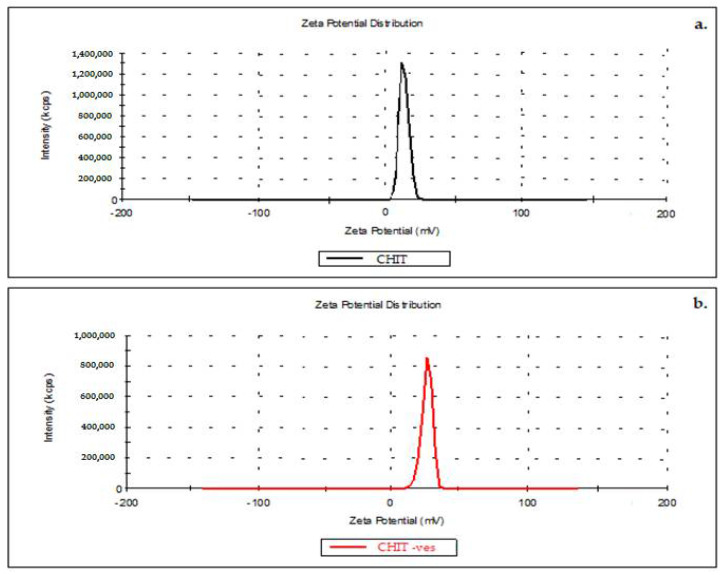
Zeta potential distribution of chitosan solution (**a**) and chitosan vesicles (**b**).

The average value of the zeta potential of 13.2 mV of chitosan solution indicates a low level of stability, as the suspension reaches the agglomeration threshold (Figure 3a).

The obtained chitosan vesicles proved to have a mean zeta potential of 32.4 mV, thus suggesting a moderate stability of the colloidal suspension (Figure 3b).

**Figure 4:**
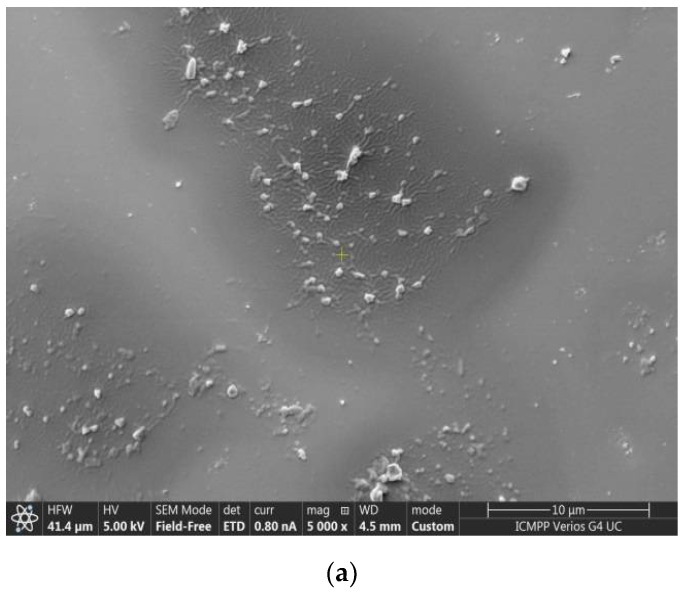

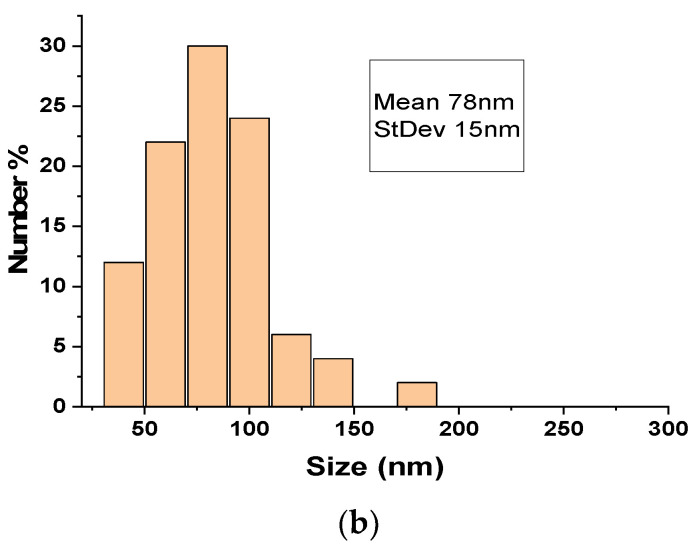
SEM micrographs (**a**) and dimensional histogram (**b**) of the chitosan vesicles.

SEM micrography (Figure 4a) indicates the presence of vesicles that tend to clump during drying on the sample holder. In SEM microscopy, the sample deposited on the support is covered with a metallic layer that protects it, so that the obtained image highlights the morphology of the analyzed surface.The dimensional histogram is made on the vesicles in the fields that do not contain agglomerations (Figure 4b).

### 3.2. Hematological Tests

There were no substantial changes regarding the number of red blood cells (RBCs) in animals from the five groupsonday 1 and day 14 of the research (Table 2). In addition, both the blood hemoglobin (Hb) and the hematocrit (Ht) levels showed no significant changes when comparing control, control alcohol, CHIT, CHIT-ves and AcA groupsonday 1 and day 14 in the experiment (Table 2).

The analysis of the leukocyte formula did not reveal statistically significant variations regarding the percentage values of PMN, Ly, E, M and B between the studied groups, namelycontrol, control alcohol, CHIT, CHIT-vesand AcA, at the specific moments of determination (Table 3).

**Figure 5:**
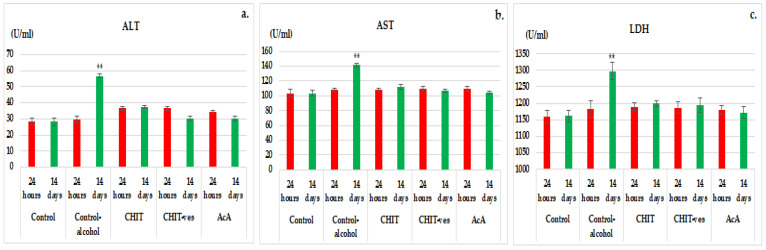
The effects of chitosan, chitosan vesiclesand ascorbic acidon the activity of ALT (**a**), AST (**b**) and LDH (**c**) in mice. The values are expressed as mean ± S.D. of the average values for 6 animals per group. ** *p* < 0.01.

After 24 h from the beginning of the experiment, a slight increase in serum ALT levels was observed in the control alcohol, CHIT, CHIT-ves and AcA groups, but without statistical significance vs. control (Figure 5a). The examination of blood collected from controlalcohol miceshowed a statistically significant increase in ALT levels (56.67 ± 6.19) (** *p* < 0.01) compared to the control group (30.29 ± 7.43) after 14 days of the experiment (Figure 5a). The ascorbic acideffects on ALT activity were more pronounced than those produced by chitosan and chitosan vesicles, but no statistical importance was noted at both 24 h and 14 days of the experiment (Figure 5a). The use of chitosan vesicleswas accompanied by a more intense decrease in the blood ALT levels than that observed for chitosan, but with a lack of statistical relevance at the two time points. Laboratory data did not identify substantial variations in ALT activity in the CHIT or AcA group compared to the control group after 14 days (Figure 5a).

A minor, but statistically insignificant, intensification in AST activity was found in all groups of tested micecompared to control after 1 day (Figure 5b). Administration of alcohol to control mice resulted in a markedly significant increase in AST activity (141.29 ± 10.37) (** *p* < 0.01) compared to the control group that did not receive alcohol (106.86 ± 9.25) onday 14 in the experiment (Figure 5b). The performed investigations did not show major differences in AST values in the CHIT, CHIT-ves or AcA group vs. the control group at the last time point of determination (Figure 5b). Even if the influence of ascorbic acidon the AST levels was higher than that of chitosanand chitosan vesicles, this fact was not statistically important at both moments of the determination (Figure 5b). The treatment with chitosan vesicleswas associated with a more intense impact on AST activity than chitosanbut without statistical significance during the experiment.

Analyzing the values obtained for serum LDH at 24 h, we noted an unimportant increase in the control alcohol, CHIT, CHIT-ves and AcA groups after 24 h (Figure 5c). In the control alcoholgroup a substantial increase (** *p* < 0.01) in LDH blood levels (1297.27 ± 35.43) compared to the control group (1193.85 ± 29.37) was registered after 14 days of the experiment (Figure 5c). No momentous changes in LDH activity were observed in the CHIT, CHIT-ves or AcA group by comparison withthe control group after 14 days of the experiment (Figure 5c). The ascorbic acid effects on LDH values in the blood were greater than the effectsof chitosanand chitosan vesicles, at both time points in the experiment (Figure 5c). The administration of chitosan vesicles resulted in an evident diminishing in LDH blood levels, but with a lack of statistical relevance at both time points of investigation.

**Figure 6:**
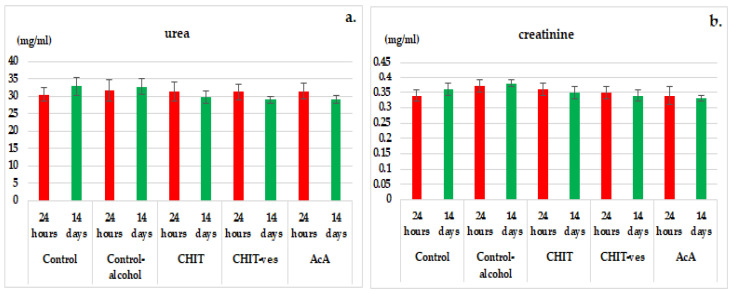
The effects of chitosan, chitosan vesiclesand ascorbic acid on the serum urea (**a**) and creatinine (**b**) levels in mice (mean ± S.D. of the average values—groups of 6 animals).

No statistically significant variations in serum urea and creatinine levels were observed in the CHIT, CHIT-ves or AcA group compared to control animals with or without alcohol, neither after 24 h nor after 14 days of the experiment (Figure 6a,b).

**Figure 7:**
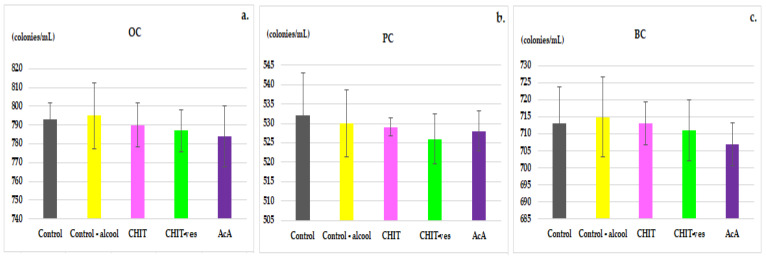
The effects of chitosan, chitosan vesiclesand ascorbic acid on the blood levels of OC (**a**), PC (**b**) and BC (**c**) in mice. The values are expressed as mean ± S.D. of the average values for 6 animals per group.

OC, PC and BC levels displayed no statistically significant differences in the CHIT, CHIT-ves or AcA group compared to non-alcoholic and alcoholic control animals at the end of the experiment (Figure 7a–c).

**Figure 8:**
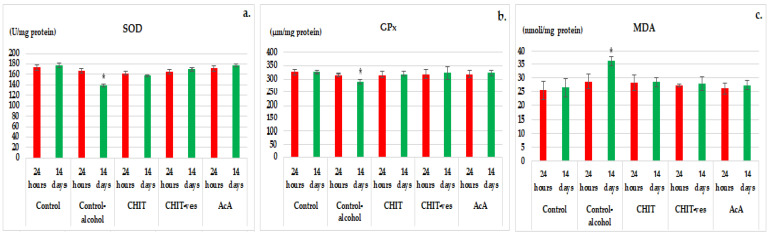
The effects of chitosan, chitosan vesicles and ascorbic acid on SOD (**a**), GPX (**b**) and MDA (**c**) activity in mice (mean ± S.D. of the average values—groups of 6 animals * *p* < 0.05).

At 24 h, a small decrease in the SOD levels was noted in the control alcohol, CHIT, CHIT-ves and AcA groups, but it was statistically irrelevant vs. control (Figure 8a). In the control alcohol group, there was a marked reduction in SOD values (138.12 ± 16.67) that was statistically significant (* *p* < 0.05) compared to the control group (169.67 ± 19.23) after 14 days ofthe experiment (Figure 8a). The effects of ascorbic acidon SOD activity were more intense than those produced by chitosanat both time moments set in the experiment (Figure 8a). The administration of chitosan vesiclesinduced more powerful effects on the serum SOD activity than chitosan, but without statistical importance after 24 h and 14 days (Figure 8a). At the end of the study, no sizeable dissimilarities in SOD levels in the CHIT, CHIT-ves or AcA group vs. control group were distinguished (Figure 8a).

At the first moment of the investigation, a reduction in GPx values in the blood was observed in all the groups tested, without high statistical relevance compared to the control group (Figure 8b). The addition of alcohol to drinking water in control mice resulted in a statistically significant decrease in GPx levels (291.17 ± 11.55) (* *p* < 0.05) compared to the control group without alcohol (321.67 ± 13.55) after 14 days of the experiment (Figure 8b). The action of ascorbic acid on GPx activity was significantly more pronounced than that of chitosanat both times in the experiment (Figure 8b). The use of chitosan vesicles was followed by a most accentuated influence on the GPx blood values, but without significance vs. the CHIT group (Figure 8b). The performed investigations did not reveal substantial variations in GPx activity inthe CHIT, CHIT-ves or AcA group compared to the non-alcoholic control group at the last time point in the experiment (Figure 8b).

After 24 h, a minor increase in MDA values was observed in the control alcohol, CHIT, CHIT-ves and AcA groups, but it was statistically insignificant vs. control (Figure 8c). Alcohol consumption in the control group showed a significant intensification (* *p* < 0.05) of MDA activity (36.17 ± 5.33) compared to the control group without alcohol (27.84 ± 4.67) after 14 days ofthe experiment (Figure 8c). No substantial differences in plasmatic MDA levels were noted in mice receiving chitosan, chitosan vesicles or ascorbic acid compared to non-alcoholic controls at the same moment of the evaluation (Figure 8c). The effects of ascorbic acidon serum MDA values were stronger than those of chitosanand chitosan vesiclesafter 24 h and after 14 days (Figure 8c). The treatment with chitosan vesiclesmanifested a more intense impact on the MDA activity than chitosan, but without marked significance during the experiment (Figure 8c).

### 3.3. Histopathology Tests

**Figure 9:**
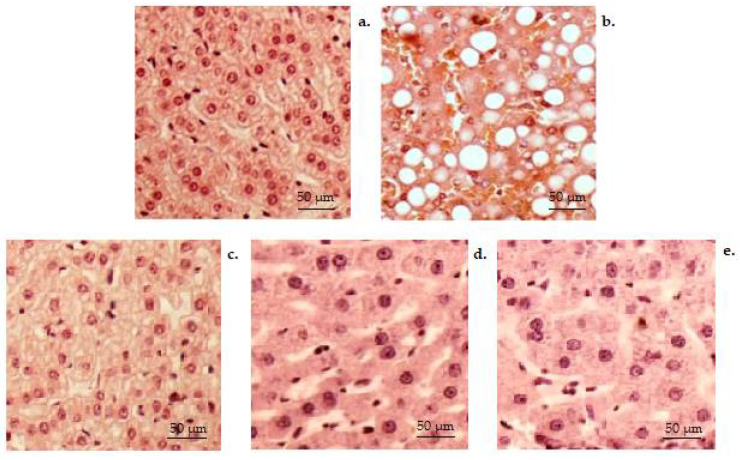
Histopathological images of the hepatic architecture in the control (**a**), control alcohol (**b**), CHIT (**c**), CHIT-ves (**d**) and AcA (**e**) groups (H&E stain × 20).

Histopathology investigation conducted on the liver tissues of animals from the non-alcoholic control group revealed a normal hepatic structure (Figure 9a). Regarding the hepatic parenchyma of the control group, a stroma with a very delicate network of collagen fibers is highlighted. A few collagen fibers are observed surrounding the central veins, in the portal area and at the level of the capsules (Figure 9a). In the animals from the control alcohol group, the hepatic histopathological analysis revealed visible alterative manifestations of the structure, with the presence of micro- and macro-vesicular steatosis, as well as multiple areas with abundant inflammatory infiltrates. There is the presence of vacuoles (triglyceride drops) in liver cells that appear deformed, and the presence of a lymphoplasmacytic infiltrate in the port and periportal space, in one or more porto-biliary spaces (Figure 9b). No considerable modification in the normal liver conformation hasbeen identified in CHIT group (Figure 9c). In CHIT-ves (Figure 9d) and AcA group (Figure 9e), histopathological analysis shows a tendency of reduction in inflammatory processes and their distribution in the liver, therefore highlighting moderate inflammation at the perivascular level.

**Figure 10:**
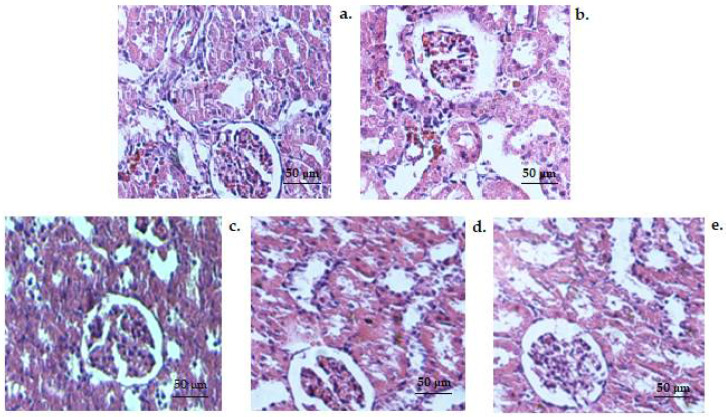
Histopathological images of the kidney structure in the control (**a**), control alcohol (**b**), CHIT (**c**), CHIT-ves (**d**) and AcA (**e**) groups (H&E stain × 20).

Microscopic examination of preparations obtained from kidney fragments taken from animals in the control group revealed a normal histological structure, characterized by the presence of glomeruli, tubules, interstitial space and blood vessels with unmodified architecture (Figure 10a). No substantial alteration in the kidney structure elements was detected in the control alcohol (Figure 10b), CHIT (Figure 10c), CHIT-ves (Figure 10d) and AcA (Figure 10e) groups compared with control animals (Figure 10a).

## 4. Discussion

In the present study, original vesicles containing chitosan were designed; the mean size of vesicles was 84 nm and the mean zeta potential was +13.2 mV, indicating a low stability of the colloidal dispersion. We can conclude that all vesicle systems were mainly positively charged regarding the zeta potential distribution, after measuring it in the colloidal solution with chitosan lipid vesicles. Following the measurement of the zeta potential, we could appreciate the fact that the colloidal solution containing lipid vesicles with chitosan has a low level of stability, being at the agglomeration threshold. Using the research technique of zeta potential determination for nanosystem characterization, a further evaluation of the solution regarding surface electric charge and definition of physical stability can be conducted [55]. Nanodispersion may present proper stability properties indicated through the high positive or negative value of the zeta potential, which further appears as a result of the electrostatic repulsion among individual particles [56]. Zeta potential values between −30 mV and+30 mV are considered to provide sufficient repulsive force for good values of the physical stability of the colloidal dispersions involved. On the other hand, a small zeta potential value plus van der Waals forces among the particles can lead to physical processes such as aggregation and/or flocculation, which arethe main cause of physical instability [57].

Our results are concordant with the literature data, revealing that the coating of vesicles with the polymer chitosan results in an improvement inthe stability of the colloidal solution. The appearance of specific charges, useful in preventing the drug release, isresponsible for the increased stability of the obtained solution [58]. The mobility of lipid molecules can be decreased both by electrostatic interaction and the existence of hydrophobic forces between chitosan and the lipid bilayer [59]. Moreover, due to the chemical properties of the polymer (positive surface charge), chitosan can selectively accumulate in affected tissue or tumors (in the case of hepatocellular carcinoma) instead of normal tissular areas after binding to biological membranes that are negatively charged [60,61,62]. Nevertheless, lipidic vesicles incorporating chitosan protect against biodegradation and interfere positively with pharmacokinetics by enhancing bioavailability [63]. Along with anticancer activities, chitosan nanoparticles demonstrated hepatoprotective activity in rats after increasing albumin levels while decreasing bilirubin, ALT and AST [57]. Furthermore, chitosan nanoparticles reduced lipid peroxidation and modulated apoptosis [62]. As the use of chitosan derivates is expanding [64], more information is essential regarding its biological properties and potential to overcome adverse reactions toparticular substances, such as alcohol.

The performed investigations in laboratory animals have shown no substantial dissimilarities in the number of GRs, Hb levels or Ht values in animals receiving chitosan, chitosan vesiclesand ascorbic acidcompared to control groups without alcohol or with alcohol in the experiment.

The leukocyte formula values (PMN, Ly, E, M, B) in the animals that received chitosan, chitosan vesiclesor ascorbic acidwere similar to the control groups both with and without alcohol in the experiment.

The treatment with chitosan, chitosan vesicles and ascorbic acid in mice with experimentally induced alcohol hepatic toxicity resulted in a statistically significant reduction inALT, AST and LDH activity compared to the control alcohol group after 14 days ofthe experiment. The effects of chitosanon the liver enzyme activity were lower than those induced by ascorbic acid in mice with alcoholic liver toxicity.

The intake of alcohol was not accompanied by notable modifications in urea and creatinine serum levels of mice treated with chitosan, chitosan vesicles or ascorbic acid, compared with alcoholic and non-alcoholic control groups.

No significant changes in immune system activity in the studied groups of mice with alcoholic liver toxicity compared to non-alcoholic animals were noted.

The use of chitosan, chitosan vesicles and ascorbic acid has been shown to reduce the oxidative stress associated with alcoholic liver toxicity. The effects of chitosanon decreasing oxidative stress in mice with alcoholic liver toxicity were weaker than those produced by ascorbic acid under the same experimental conditions.

Administration of chitosan, chitosan vesiclesand ascorbic acid has resulted in amelioration of hepatic structural impairment after alcohol consumption in mice. No significant changes in the kidney architecture among CHIT, AcA, control alcoholic and control non-alcoholic groupswere noted.

The protective effect of chitosan on hepatocytes has been investigated by researchers in the last ten years, after inducing toxic liver injury with various toxic substances and treating the laboratory animals with chitosan, either free or as nanoparticles [65,66,67,68]. Experimental studies have shown that chitosan has hepatoprotective effects due to improved endogenous antioxidant defense systems and reduced lipid peroxidation in the experimental model of toxic hepatopathy caused by acetaminophen in rats [65]. In rats with liver toxicity induced by repeated administration of carbon tetrachloride, chitosan displayed protective and antioxidant activity characterized by an attenuation ofthe blood biochemical disturbances and the histological alterations evidenced in the liver tissue [66,67]. In an experimental investigation, the researchers proved that the use of titanium (IV)–dithiophenolate complex chitosan nanocomposites decreases the oxidative stress marker activity and improves the functional and structural liver injuries produced by carbon tetrachloride in rats [68].

Other studies have investigated the effects of chitosanonliver tissue regeneration in rats with liver injury induced by thioacetamide [69]. Chitosan had not only curative and proliferative effects but also anti-inflammatory action, decreasing TNF-α levels in laboratory animals and improving hepatic and renal function as well as the structural alterations in animals with thioacetamide-induced liver damage [69].

The hepatoprotective effects of chitosan and chitosan nanoparticles were also noted in liver injuries induced by various hepatotoxic substances, such as diethylnitrosamine in rats [70] and emamectin benzoate in mice [71]. The rats treated with chitosan had reduced levels of serum liver marker enzymes and increased activity of antioxidant parameters and of serum albumin in animals with diethylnitrosamine-induced liver toxicity [70]. The use of chitosan and chitosan nanoparticles amended the blood biochemical modifications, reduced the oxidative stress, decreased the expression of some specific genes in liver with a decreasein elevated DNA damage and improved the structural hepatic alterations [71].

Other researchers obtained chitosan nanoparticles based on the ion gelation method which displayed protective properties on 2-nitropropane-induced hepatic injury in rats, demonstrated by the amelioration of the functional biochemical parameters and the associated oxidative stress, as well as by the decrease in inflammatory markers and the restoration of normal liver conformation [6].

Several studies have focused on the perspectives of preventing or treating iatrogenic hepatotoxicity by co-administration of chitosan supplements. An experimental studyconducted by Santosh et al. investigated liver protection in rats treated with isoniazid and rifampicin, antitubercular drugs that induce hepatic side effects. Chitosan significantly prevented liver alterations and reduced the levels of marker enzymes in rats treated with both substances [72].

The study was based on the idea of using innovative chitosan nanoparticles to investigate their influence on hematological, biochemical and immunological parameters and on oxidative stress in mice with alcoholic liver disease. Chitosan is a cationic polysaccharide that is soluble in gastric acid, which greatly reduces its bioavailability and thus its pharmacodynamic effects. The design of nanoparticles with chitosan and phosphatidylcholine and the original method used for their production offer the possibility to improve these parameters. Moreover, this production procedure may be a starting point for the incorporation of drugs for biomedical applications into soft lipid vesicles, due to their improved properties, especially in terms of prolonging the release time of the active substance.

The results of our research are limited by both the reduced period of time for chitosanadministration and the number of animals used during the experiment and should therefore be reinforced by future studies.

## 5. Conclusions

Laboratory analysis did not show significant differences in blood GR values, Hb levels or Ht values or the percentage values of the leukocyte formula elements or significant changes in the immune system activity between the four tested groups and the control group.

In the animals from the control alcohol group, the hepatic histopathological analysis revealed visible alterative manifestations of the structure, but no considerable modification in the normal liver conformation was identified in the CHIT group. In the CHIT-ves and AcA groups, histopathological analysis shows a tendency of a reduction in inflammatory processes and their distribution in the liver.

No substantial alteration in the kidney structure elements was detected in the control alcohol, CHIT, CHIT-ves or AcA group compared with control animals. In our experimental conditions, the use of chitosan improved the liver functional and structural damages and decreased the oxidative processes in alcoholic mice.

## Figures and Tables

**Table 1:** Experimental protocol to evaluate the effects of chitosan vesicles in mice with alcohol-induced liver toxicity.

Investigation Steps	Day of Experiment													
	1	2	3	4	5	6	7	8	9	10	11	12	13	14
Control (water *ad libitum*)														
5% alcohol in water *ad libitum*														
Control alcohol														
CHIT														
CHIT-ves														
AcA														
5 g/kg orally of 10% alcohol solution														
Blood collection														
Liver fragment collection														

**Table 2:** The effects of chitosan, chitosan vesiclesand ascorbic acidon the number of RBCs and blood values of Hb and Ht in mice (mean ± S.D. of the average values—groups of 6 animals).

Groups	Time Points of Determination	RBCs (mil/μL)	Hb (g/mL)	Ht (%)
Control	24 h	8.47 ± 1.60	13.79 ± 2.29	38.35 ± 6.09
	14 days	8.55 ± 0.98	14.07 ± 2.41	38.22 ± 5.67
Control alcohol	24 h	8.41 ± 1.33	14.37 ± 2.17	38.12 ± 5.39
	14 days	7.79 ± 1.06	13.72 ± 2.11	39.19 ± 6.33
CHIT	24 h	8.28 ± 2.09	13.44 ± 2.43	37.64 ± 5.81
	14 days	8.45 ± 1.43	13.58 ± 2.67	37.42 ± 5.43
CHIT-ves	24 h	8.34 ± 1.15	13.97 ± 2.81	38.24 ± 5.29
	14 days	7.97 ± 1.37	13.19 ± 1.33	37.79 ± 5.55
AcA	24 h	8.12 ± 1.67	14.48 ± 3.21	38.72 ± 6.13
	14 days	8.24 ± 1.55	14.13 ± 2.45	38.47 ± 5.37

**Table 3:** Leukocyte formula (neutrophilic polymorphonuclear cells—PMN, lymphocytes—Ly, eosinophils—E, monocytes—M, basophils—B) in CHIT, CHIT-ves and AcA groups (mean ± S.D. of the average values—groups of 6 animals).

Groups	Time points of Determination	Leukocyte Formula Elements (%)				
		PMN	Ly	E	M	B
Control	24 h	14.26 ± 6.59	83.22 ± 9.03	0.45 ± 0.17	1.78 ± 1.23	0.29 ± 0.15
	14 days	14.55 ± 7.48	82.61 ± 9.11	0.51 ± 0.27	2.05 ± 1.81	0.28 ± 0.19
Controlalcohol	24 h	13.67 ± 7.11	83.77 ± 9.23	0.49 ± 0.11	1.78 ± 1.23	0.29 ± 0.13
	14 days	13.43 ± 6.72	83.74 ± 9.13	0.48 ± 0.15	2.05 ± 1.81	0.30 ± 0.37
CHIT	24 h	13.36 ± 7.41	83.88 ± 8.07	0.47 ± 0.13	2.09 ± 0.67	0.22 ± 0.13
	14 days	13.52 ± 7.55	84.17 ± 8.12	0.50 ± 0.11	1.56 ± 0.43	0.25 ± 0.11
CHIT-ves	24 h	14.15 ± 7.26	83.81 ± 8.07	0.50 ± 0.23	1.23 ± 1.17	0.31 ± 0.25
	14 days	13.09 ± 8.48	85.84 ± 8.12	0.49 ± 0.17	1.03 ± 0.56	0.33 ± 0.57
AcA	24 h	14.84 ± 8.33	82.29 ± 9.21	0.48 ± 0.13	2.07 ± 1.03	0.32 ± 0.19
	14 days	14.52 ± 8.07	82.57 ± 8.33	0.46 ± 0.21	2.15 ± 1.26	0.30 ± 0.11

## Data Availability

Not applicable.

## Abbreviations

alanine aminotransferase	ALT
ascorbic acid (group)	AcA
aspartate aminotransferase	AST
bactericidal capacity of peritoneal macrophages	BC
basophils	B
chitosan (group)	CHIT
chitosan vesicles (group)	CHIT-ves
eosinophils	E
glutathione peroxidase	GPx
hemoglobin	Hb
hematocrit	Ht
lactate dehydrogenase	LDH
lymphocytes	Ly
malondialdehyde	MDA
monocytes	M
peripheral blood	PC
polymorphonuclear neutrophils	MN
serum opsonic capacity	OC
superoxide dismutase	SOD
